# SMN protein promotes membrane compartmentalization of ribosomal protein S6 transcript in human fibroblasts

**DOI:** 10.1038/s41598-020-76174-3

**Published:** 2020-11-04

**Authors:** Francesca Gabanella, Annalisa Onori, Massimo Ralli, Antonio Greco, Claudio Passananti, Maria Grazia Di Certo

**Affiliations:** 1 CNR-Institute of Biochemistry and Cell Biology, Department of Sense Organs, Sapienza University of Rome, Viale del Policlinico, 155-00161 Rome, Italy; 2 CNR-Institute of Molecular Biology and Pathology, Department of Molecular Medicine, Sapienza University of Rome, Viale Regina Elena, 291-00161 Rome, Italy; 3 Department of Sense Organs, Sapienza University of Rome, Viale del Policlinico, 155-00161 Rome, Italy

**Keywords:** Biochemistry, Cell biology, Molecular biology

## Abstract

Alterations of RNA homeostasis can lead to severe pathological conditions. The Survival of Motor Neuron (SMN) protein, which is reduced in Spinal Muscular Atrophy, impacts critical aspects of the RNA life cycle, such as splicing, trafficking, and translation. Increasing evidence points to a potential role of SMN in ribosome biogenesis. Our previous study revealed that SMN promotes membrane-bound ribosomal proteins (RPs), sustaining activity-dependent local translation. Here, we suggest that plasma membrane domains could be a docking site not only for RPs but also for their encoding transcripts. We have shown that SMN knockdown perturbs subcellular localization as well as translation efficiency of RPS6 mRNA. We have also shown that plasma membrane-enriched fractions from human fibroblasts retain RPS6 transcripts in an SMN-dependent manner. Furthermore, we revealed that SMN traffics with RPS6 mRNA promoting its association with caveolin-1, a key component of membrane dynamics. Overall, these findings further support the SMN-mediated crosstalk between plasma membrane dynamics and translation machinery. Importantly, our study points to a potential role of SMN in the ribosome assembly pathway by selective RPs synthesis/localization in both space and time.

## Introduction

The RNA life cycle is accomplished through distinct phases promoting synthesis, modifications, traffic, and translation^1–3^. Each of these phases takes place in a specific subcellular location, arguing that “where”, and not only “how”, these processes occur, can be crucial for RNA homeostasis^4^. Cytoplasmic touring of individual mRNAs coupled with localized translation may ensure a common mechanism regulating gene expression at the subcellular level^5–8^. A large fraction of mRNAs is actively trafficked to subcellular compartments by association with trans-acting RNA binding proteins (RBPs) and assembly into ribonucleoprotein particles (mRNPs)^5^. Moreover, several studies highlight an intricate link between RNA metabolism and membrane trafficking. The coatomer protein I (COPI) vesicle complex, which moves cargos within the Golgi-ER network, has been described as a conserved mechanism of RNPs trafficking^9–11^. Destruction of the COPI pathway results in RNA mis-localization in yeast^12^. In addition, the alpha subunit of the COPI complex moves to the cell periphery with specific mRNAs, whose molecular motifs identify mainly plasma membrane- and cytoskeleton-related pathways^12^. Several findings suggest that the plasma membrane may contribute to compartmentalize protein synthesis in subcellular sites. In highly polarized cells, the plasma membrane binds to and actively releases components of translation machinery for spatially restricted protein production^13,14^. Endosomes, which are known to internalize cargos from the plasma membrane, can operate also as shuttles for subcellular delivery of mRNPs^15,16^. Accordingly, it has been demonstrated that RNPs-bearing endosomes dock at mitochondria and provide a platform for compartmentalized protein synthesis^17^. It is therefore reasonable to suppose that membrane dynamics may influence RNA homeostasis as well as mRNA translation underlying specialized subcellular activities. In this framework, RNA-related proteins may be key determinants dictating the proteome profile in time and space.

Low levels of the Survival Motor Neuron (SMN) protein cause Spinal Muscular Atrophy (SMA), a neurological disease leading to infant mortality^18,19^. Clinical manifestation of SMA reflects degeneration of motor neurons in the brain stem and spinal cord. Although SMA is widely known as a motor neuron disease, additional organs may be perturbed by SMN deficiency, especially in the most severe forms of the disease. This is consistent with the notion that SMN is an essential protein whose loss becomes deleterious in all cell types^20–25^. SMN plays a key role in the RNA life cycle. It is required for resolving transcription termination regions^26^. It facilitates the assembly of distinct RNPs, such as small nuclear RNPs (snRNPs), small nucleolar RNPs (snoRNPs), and small Cajal body RNPs (scaRNPs)^27,28^ and it promotes assembly and trafficking of messenger ribonucleoprotein (mRNP) complexes^29^. Furthermore, many studies support an involvement of SMN in translational control. Notably, SMN physically associates with translation machinery components^14,30^. Furthermore, we have previously found a relationship between SMN and membrane-bound ribosomal proteins (RPs). We showed that SMN coexists with RPs in caveolin-rich membrane domains and promotes spatially restricted protein production underlying membrane remodelling^14^. Importantly, SMN affects mammalian target of rapamycin (mTOR) activity, suggesting a role of SMN in cap-dependent translation^31,32^. Finally, it is important to mention that SMN might affect translation pathways early, by regulating transcripts coding for ribosomal proteins (RPs). A recent work, using HITS-CLIP methodology, identified a variety of SMN-associated RNA. Most of these RNAs were protein-coding mRNAs, including those involved in ribosome biogenesis^33^. This work agreed with a previous study showing that a FLAG-tagged SMN protein, expressed in motor neuron-like cells, associates with RP-coding transcripts^34^.

Here, we have further investigated the relationship between SMN and RP-coding transcripts. We have shown that SMN associates to and affects subcellular distribution of RPS6 mRNA. We also found that SMN knockdown perturbs the translation rate of RPS6 during active membrane and cortical actin remodelling. Interestingly, we observed that membrane compartments sequester a subset of RPS6 transcripts in an SMN-dependent manner. Moreover, we provide evidence that SMN could bridge RP-coding transcripts to caveolin-rich membrane domains. Notably, for the first time we have obtained a spatial mapping of RPS6 mRNA in single cells by using the target RNA-initiated rolling circle amplification method. Collectively, these findings confirm the intriguing relationship between membrane trafficking and the translation pathway. Furthermore, this study suggests that SMN could mediate peripheral localization of a subset of RP-coding transcripts, contributing in this way to select protein synthesis in time and space.

## Results

### Ribosomal machinery at actin-rich membrane protrusions

**Figure 1:**
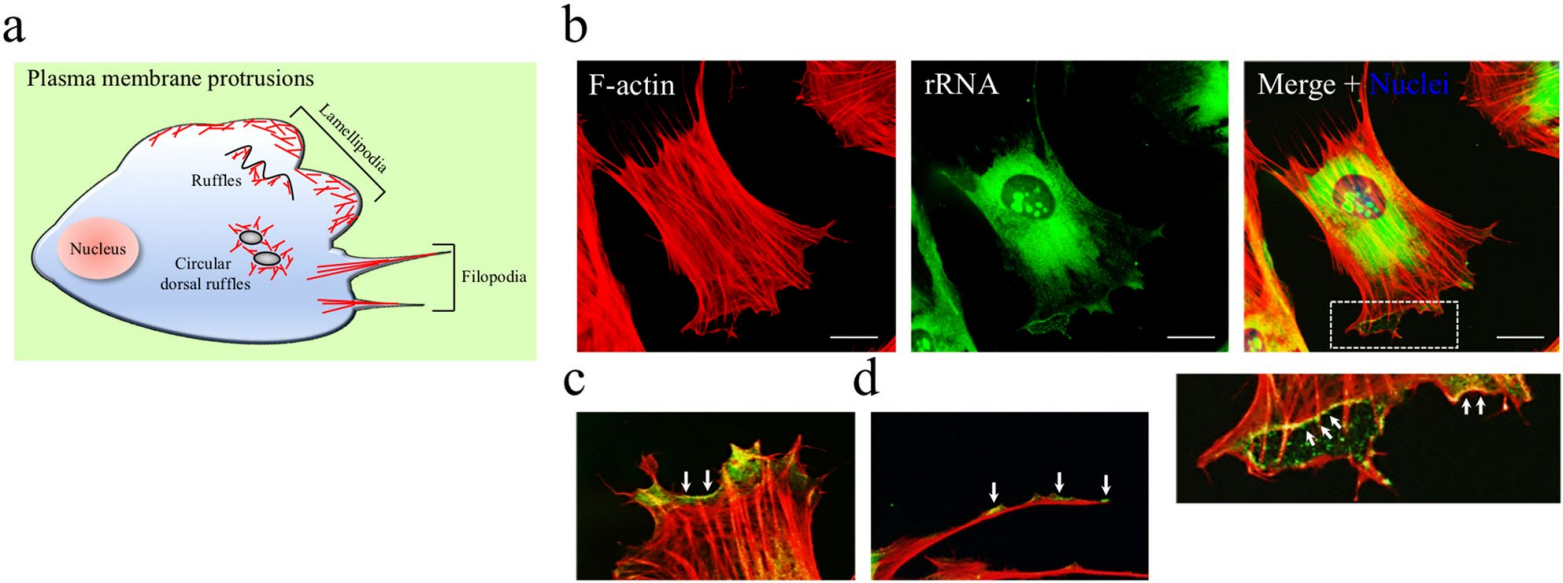
Ribosomal RNA at the plasma membrane protrusions. (**a**) The cartoon illustrates a typical fibroblast displaying actin-rich membrane protrusions. The actin filament supports the extension of the plasma membrane generating typical structures such as lamellipodia, filopodia and ruffles. (**b**) Representative fluorescence microscopy images. Human fibroblasts were stained with Alexa Fluor 594-phalloidin to visualize the actin filaments (F-actin, red). Indirect immunofluorescence using the monoclonal antibody Y10B against ribosomal RNA (rRNA, green). Nuclei were labelled with DAPI (blue, in the merge panel). Scale bar, 10 µm. The bottom panel represents a higher magnification of the boxed area. Arrows indicate rRNA dots along the perimeter of a circular dorsal ruffle. (**c**,**d**) Representative details of actin-rich membrane protrusions, such as lamellipodia (**c**) and filipodia tips (**d**), in which rRNA accumulations.

A subset of RPs resides at peripheral regions of cells/tissues, which are characterized by distinct functional specialization of their surface^13,14,35^. Although we cannot exclude extra-ribosomal functions, RPs generally indicate fully assembled ribosome and/or ribosomal subunits. Indeed, a recent study showed ribosomes aligned underneath the plasma membrane of unstimulated axonal growth cones^36^. As reported by the authors, electron microscopy analysis was consistent with monosomes binding to the intracellular domains of transmembrane receptors. To further validate the presence of ribosomal machinery at the cell periphery, we subjected human fibroblasts to an immunofluorescence assay. As schematically depicted in Fig. 1a, the fibroblast exhibits an asymmetrical cell surface behaviour revealed by typical actin-based membrane protrusions^37–39^. In this regard, the fibroblast represents a well-established model among polarized cells. First, we labelled cells with Y10B monoclonal antibody, which targets ribosomes through its immunoreactivity to ribosomal RNA (rRNA)^40^. Then, we imaged cells by fluorescence microscopy. A strong fluorescent signal was detectable in canonical locations of rRNA, such as the nucleolus and cytoplasm (Fig. 1b)^41^. In addition, Y10B immunoreactivity was also appreciable in membrane protrusions of the labelled fibroblasts (Fig. 1b). Notably, discrete dots of rRNA were clearly visualized in close proximity to the cell perimeter of specialized actin-rich domains, such as lamellipodia and ruffles (Fig. 1c). Moreover, rRNA staining was also enriched in filopodia tips (Fig. 1d). In line with other studies^36^, these results suggest that subregions of the plasma membrane may retain ribosomal complexes, and further support the idea of a polarized anchoring of translation machinery.

### SMN affects subcellular distribution of RPS6 mRNA

**Figure 2:**
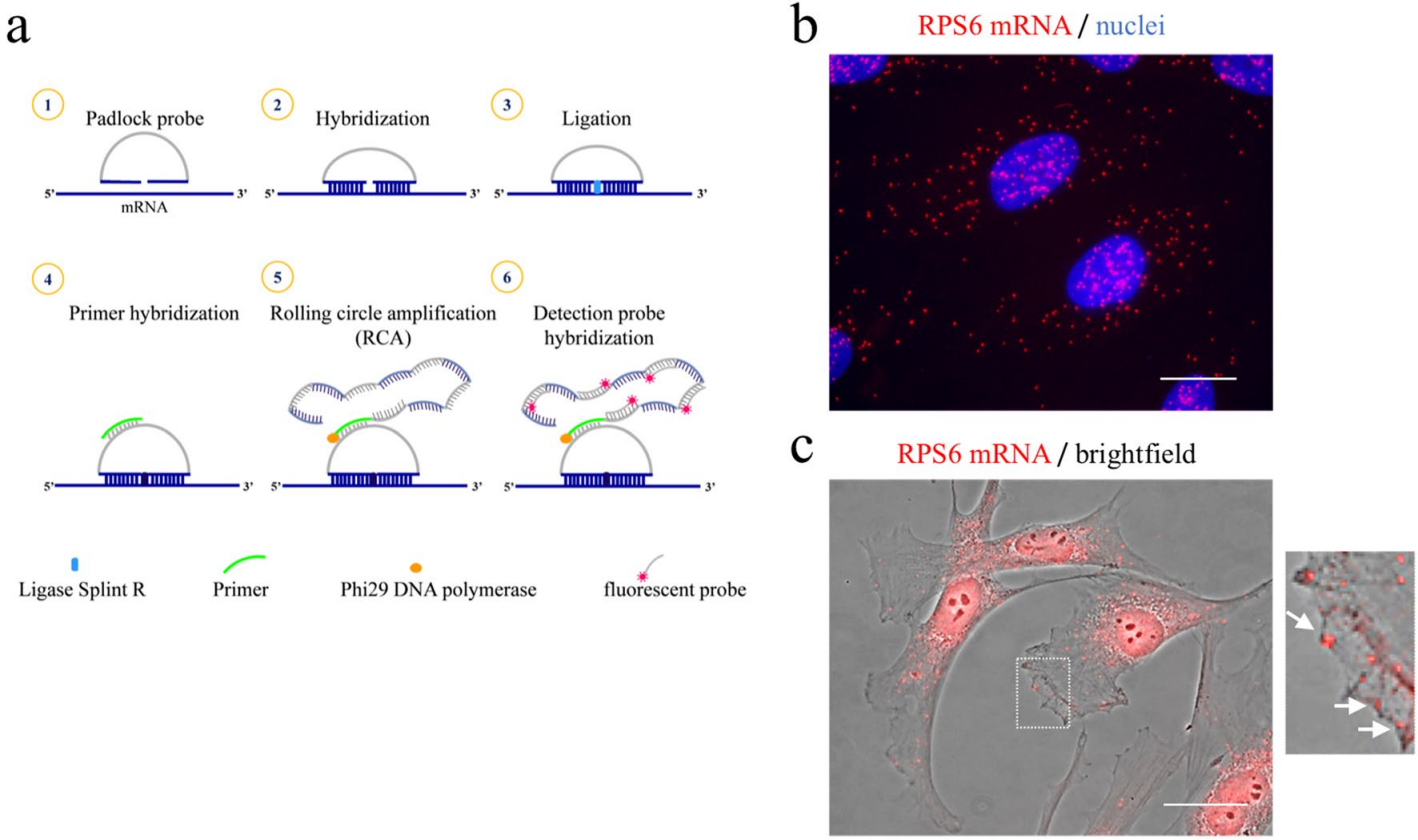
Subcellular distribution of RPS6 mRNA. (**a**) Diagram illustrating main experimental steps of the padlock assay in fixed cells. A padlock probe is designed with the 5′- and 3′-terminal bases complementary to the target sequence of mRNA of interest. By using the Splint R ligase, padlock probe is ligated and circularized with the mRNA as the template. Short DNA primer is used for initiating rolling circle amplification (RCA). In this way the target sequence is converted in a long DNA amplicon with hundreds of copies of the padlock probe. Finally, amplicons become detectable by using a fluorophore-labelled hybridization probe. (**b**) Representative images of human fibroblasts subjected to a padlock assay targeting RPS6 mRNA. AlexaFluor 595-labelled probe allowed the detection of RCA dots (red). Nuclei were labelled with DAPI (blue). Scale bars, 10 µm. (**c**) Overlapped brightfield and padlock images. Scale bars, 20 µm. Higher magnification panel reveals the presence of RCA dots closely associated to the plasma membrane.

**Figure 3:**
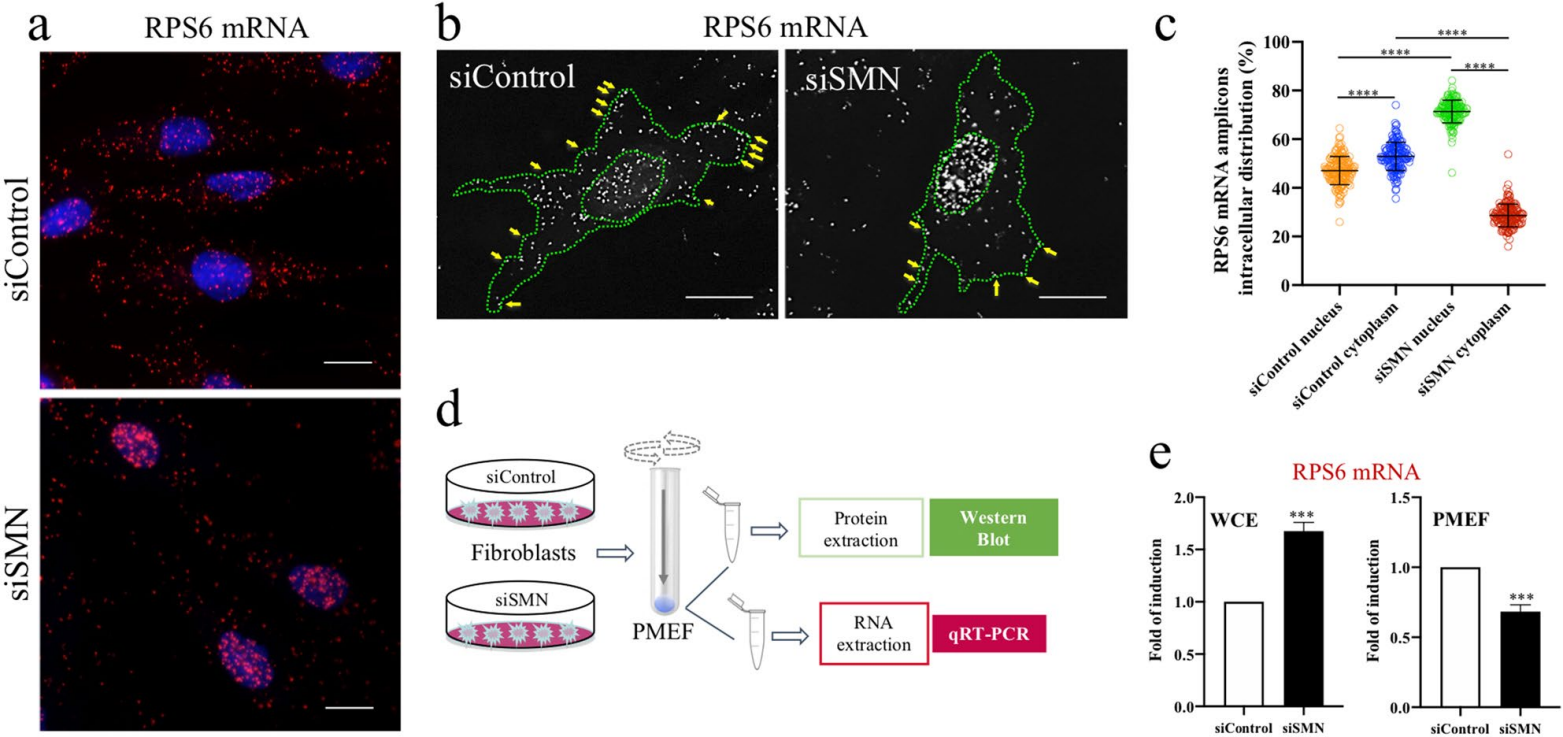
SMN perturbs intracellular sorting of RPS6 mRNA. (**a**) Padlock assay targeting RPS6 mRNA (red dots) in siControl- and siSMN-transfected fibroblasts. Nuclei were labelled with DAPI (blue). Scale bar, 10 μm. (**b**) Representative images of RPS6 mRNA distribution (white dots) in single cell. Outlines of cell surface and nucleus are marked by a green dotted line. Yellow arrows indicate the localization of RPS6 mRNA amplicons along the cell edges. Scale bar, 10 μm. (**c**) Scatterplot representing the percentage of RCA dots located within the cytoplasm outline or nuclear outline of both siControl- and siSMN-transfected fibroblasts (n = 50 cells were analysed for each condition. In graph are plotted all the results from three independent experiments; ****P < 0.0001 one-way ANOVA-Bonferroni’s multiple comparisons test). Mean ± s.d. are illustrated. (**d**) Schematic methodology to obtain the plasma membrane-enriched fraction (PMEF) from cultured cells. RNA pool of each PMEF was purified and analysed by quantitative real-time PCR (qRT-PCR). (**e**) Quantification of RPS6 transcript by qRT-PCR. For each experimental condition, both the whole cell extract (WCE) and PMEF fraction were assessed. Data are shown as fold induction comparing siSMN with siControl cells Asterisks indicate significative differences using unpaired t-test (***P < 0.01 WCE *P *value = 0.0002; PMEF *P *value = 0.0004). Data represent means from three independent experiments. Error bar indicate s.d.

The significance of ribosomal machinery at the cell surface raised some attractive questions. Can the plasma membrane sequester mRNA-containing complexes? If yes, can SMN affect membrane-bound transcripts as it does for ribosomal proteins?^14^ In this context, we started to explore transcripts encoding for structural components of translation machinery, such as ribosomal proteins. Exploring RP-coding mRNAs in subcellular compartments appeared exciting for several reasons: (1) RP-coding mRNAs have been found enriched in peripheral regions of a variety of cell types, including neurons and epithelial cells^35,42,43^; (2) it has been reported that SMN impacts translation efficiency of RP-coding transcripts^44^; finally, (3) locally synthesized ribosomal proteins may sustain asymmetrical protein synthesis in highly specialized cells^35^. First, our intent was to visualize the distribution of RP-coding transcripts in single cells. To do this, we subjected fixed cells to a padlock assay, which combines padlock probes and rolling circle amplification (RCA) (Fig. 2a). This strategy generates one-target-one-amplicon in situ amplification, allowing one to achieve highly specific imaging of a selected mRNA, with a near-single-molecule resolution^45^. We designed a padlock assay targeting the mRNA of ribosomal protein S6, a key component of the eukaryotic ribosome. Then, we imaged cells by high-resolution fluorescence microscopy. As shown in Fig. 2b, RCA dots provided a surprisingly intracellular map of RPS6 mRNA. RPS6 mRNA appeared diffusely distributed throughout the cytoplasm and nuclear/perinuclear regions of fibroblasts. Notably, several fluorescent dots were positioned at the cell periphery, and some of these clearly overlapped with plasma membrane subdomains (Fig. 2c). This distribution/frequency of RCA dots has been found in more than 80% of examined cells. Interestingly, the peripheral localization of RPS6 mRNA seems to be consistent with the notion that different subsets of mRNAs can associate to specific receptors at the plasma membrane^36^. The next step was to explore a possible relationship between the intracellular sorting of RPS6 mRNA and SMN. We first down-regulated SMN expression levels by transfection of human fibroblasts with a pool of SMN1-selective siRNAs (siSMN). Scrambled siRNAs were used as control (siControl). 48 h after transfection, fibroblasts were subjected to a padlock assay targeting RPS6 mRNA (Fig. 3a). In order to identify SMN deficient cells we combined padlock assay with SMN immunostaining (Supp. Figure S1). In our system, no fluorescence signals above background levels were detectable in approximately 90% of siSMN-transfected fibroblasts. As expected, in siControl-transfected cells, RPS6 mRNA appeared diffusely distributed throughout the cytoplasm, in both peripheral and perinuclear districts. Conversely, RPS6 mRNA dots clearly accumulated at nuclear/perinuclear subregions upon SMN depletion (Fig. 3a,b). Quantitative analysis of fluorescent dots revealed that SMN knockdown caused a slight yet significant increase of RPS6 mRNA (Supp. Figure S1). However, the higher frequency of the fluorescent dots shifted from the cytoplasm to more nuclear/perinuclear locations in SMN-deficient cells, which exhibited also a reduction of RPS6 mRNA closely associated with the cell surface (Fig. 3c). These data suggested that SMN can impact subcellular sorting of RPS6 mRNA. To further validate this notion, we carried out a biochemical method previously optimized to isolate plasma membrane-enriched fractions (PMEFs) from cultured cells^14^ (Fig. 3d). Therefore, both siControl- and siSMN-transfected fibroblasts were processed to obtain PMEFs. Each PMEF was subjected to Western Blot analysis validating both transfection efficiency and enrichment of caveolin-1, an integral membrane protein (Supp. Figure S2). Then, we proceeded with the extraction of total RNA from whole cells and their respective PMEFs. Total RNA has been recovered not only from whole cell extracts (WCE), but also from plasma membrane fractions. By a quantitative RT-PCR (qRT-PCR) we evaluated the abundance of RPS6 transcript in both the WCE and PMEF preparations. As shown in Fig. 3e, WCE graph, we found an overall up-regulation of RPS6 gene in SMN-deficient cells, compared to control cells. Despite its higher abundance in whole cells, RPS6 transcript was reduced in PMEFs from SMN-deficient cells (Fig. 3e, PMEF). Notably, this discordant result has been confirmed in primary fibroblasts of both a severe type I SMA patient and an unaffected individual (Supp. Figure S3). By these findings, we conclude that: (1) the plasma membrane could be a docking site for mRNA-containing complexes; and, most importantly, (2) SMN could promote anchoring not only of ribosomal proteins but also of their encoding mRNA. Given these encouraging results, discovering transcriptional signature of plasma membrane-derived fractions in healthy as well as SMA-affected cells, will represent our priority. Of note, to our knowledge, this is the first study visualizing intracellular localizations of endogenous RPS6 mRNA.

### SMN associates to and affects translation efficiency of RPS6 mRNA

**Figure 4:**
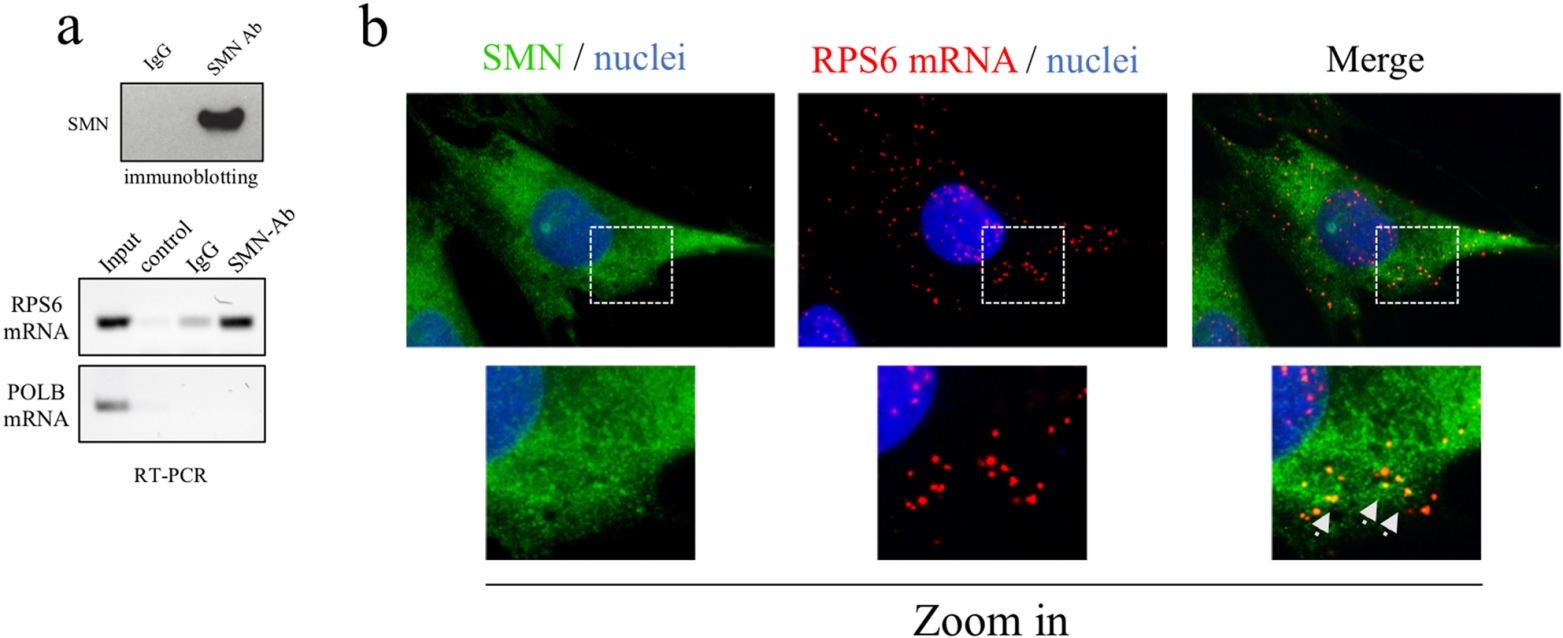
SMN binds to RPS6 mRNA. (**a**) Cellular extracts were processed for RNA-immunoprecipitation (RIP) assay using SMN monoclonal antibody-conjugated beads. As negative control was used mouse IgG-conjugated beads. (upper panel) Immunoblotting validating the efficiency of SMN immunoprecipitation. (bottom panel) RPS6 mRNA and DNA Polymerase Beta (POLB) mRNA in RIP samples was checked by semiquantitative RT-PCR analysed by agarose gel electrophoresis. Panels are representative of three independent experiments. Uncropped gels/blots are displayed in supplementary information (**b**) Fibroblasts were subjected to a combination of SMN immunostaining (green) and padlock assay targeting RPS6 mRNA (red), and then were imaged by epifluorescence microscopy. Representative images showing overlapped fluorescent signals (yellow dots). Nuclei were stained with DAPI (blue). Scale bar, 10 µm. Higher magnification of the boxed area reveals some of the overlapped dots in close proximity to the cell surface (arrows).

**Figure 5:**
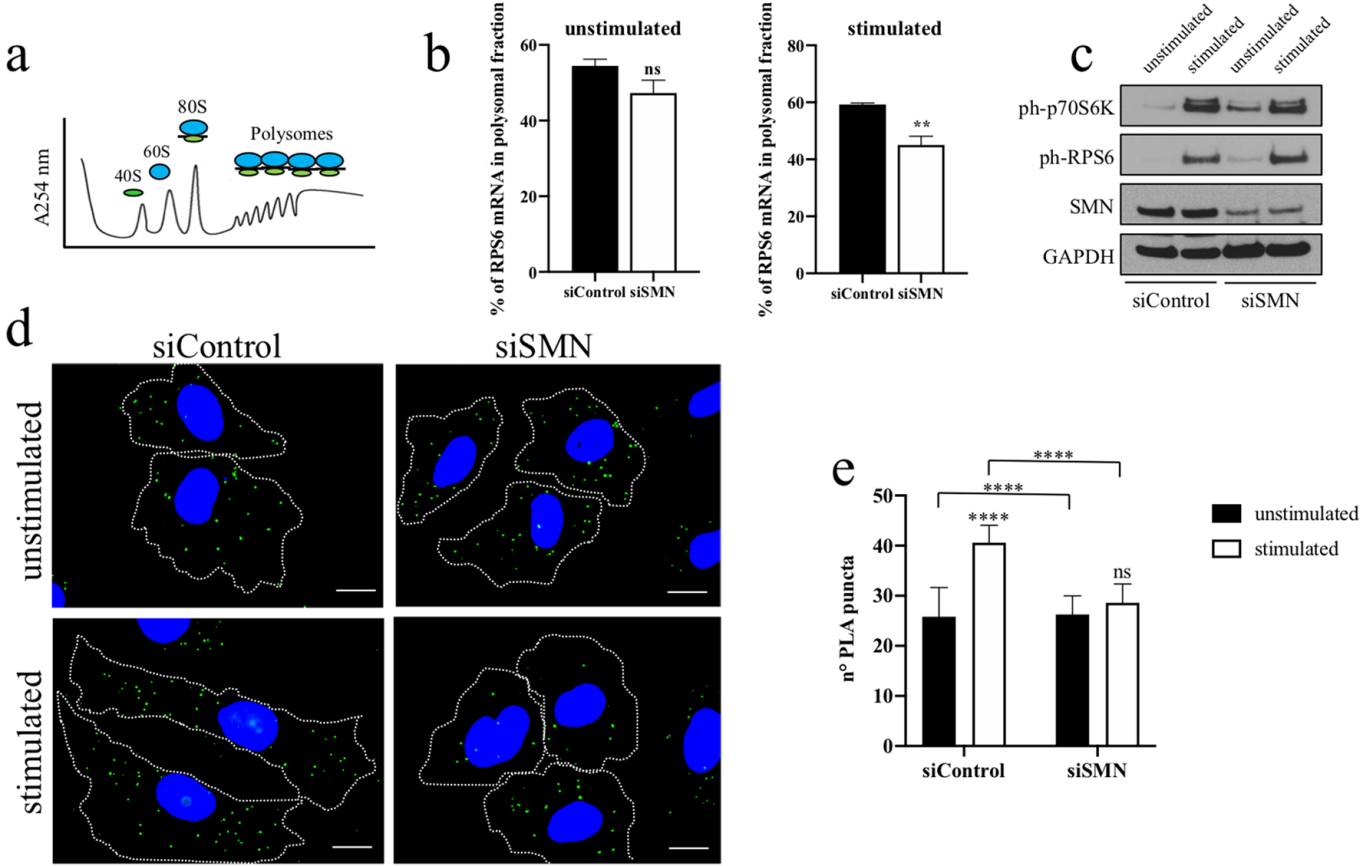
SMN impacts activity-dependent translation of RPS6 mRNA. (**a**) Graphical representation of the absorbance peaks at 254 nm in a typical polysome profile. The position of fractions corresponding to 40S, 60S, 80S monosomes, and polysomes is indicated. (**b**) Quantification by qRT-PCR of RPS6 mRNA abundance in polysomal fractions from unstimulated or not, transfected fibroblasts. The graph illustrates the mean of three independent experiments. Data are reported as a percentage of total RNA. Error bars represent s.d., Asterisks indicate significative differences using unpaired t-test (** *P* < 0.01; *P *value = 0.0014; ns = not significant). (**c**) Western blot analysis of siControl and siSMN-transfected fibroblasts, stimulated or not by ATP depletion/recovery assay. Equal amounts of proteins were immunoblotted for ph-p70S6K (Thr389), ph-RPS6 (Ser235 or Ser236) and SMN. GAPDH was monitored as control of the protein loading. Uncropped blots are displayed in supplementary information (**d**) Puro-PLA assay visualizing subcellular localizations of newly synthesized RPS6 protein. Puro-PLA assay was carried out using primary antibodies against the puromycin and RPS6, monoclonal and polyclonal, respectively. Both siControl- and siSMN-transfected fibroblasts were stimulated or not by ATP depletion and recovery treatment. Detection of Puro-PLA signals (green dots) was achieved by epifluorescence microscope. Nuclei were stained with DAPI (blue). Scale bars, 10 µm. (e) Quantification of Puro-PLA puncta per cell. n = 50 cells were analysed for each condition. ****P < 0.0001 one-way ANOVA-Bonferroni’s multiple comparisons test; ns = not significant). Data are the mean from three independent experiments. Error bars indicate s.d.

We asked whether SMN could impact additional aspects within the metabolism of RPS6 mRNA. First, by RNA-immunoprecipitation (RIP) experiments, we showed a physical link between SMN and RPS6 mRNA (Fig. 4a). This result was in line with a previous work reporting that FLAG-SMN chimeric proteins co-precipitate with RP-coding transcripts^34^. Coherently, immunofluorescence analysis revealed a partial overlapping of SMN immunostaining and RCA dots generated by padlock assays targeting RPS6 transcripts (Fig. 4b). Next, given the role of SMN in translational control, we verified a possible involvement of SMN in the synthesis of ribosomal S6 protein. We previously reported that SMN knockdown preferentially perturbs activity-dependent translation^14^. We suggested that a subset of protein synthesis, downstream of mTOR and underlying cytoskeleton remodelling, depend on SMN function. We suspected that transcripts translationally affected by SMN could include the mRNA of RPS6. To test this hypothesis, we used a polysome profile analysis. This method allows one to obtain two types of transcripts: (1) repressed/poorly translated transcripts, associated with ribosomal subunits/monosomes, and (2) efficiently translated transcripts, which are associated with polysomes^46^. Cytoplasmic extracts were stratified by ultracentrifugation on sucrose density gradients. Fractions, from lightest to heaviest, were collected and identified by continuous measurement of absorbance at 254 nm. Graphical representation of a typical polysome profiling is shown in Fig. 5a. The first peak contains free cytosolic ribonucleoproteins (RNPs), and the subsequent peaks include ribosomal subunits (40S and 60S) and monosomes (80S), all associated with non-translating complexes. The remaining peaks of the profile include materials that sediment with high sucrose concentrations and contain mRNAs associated with translating polysomes. Both siControl- and siSMN-transfected fibroblasts were processed for polysome profiling. Furthermore, experiments were conducted under basal and stimulating conditions. We stimulated fibroblasts by ATP depletion and recovery treatment. As previously reported, this method triggers distinct but overlapping pathways supported by SMN functions, such as local translation and actin remodelling^14,47,48^. Therefore, we compared polysomal profiles from SMN-deficient and control cells. As revealed by absorbance peaks, changes were appreciable in stimulating conditions only, where the overall translation rate shifted toward a moderate repression upon SMN knockdown (Supp. Figure S4). To monitor specifically the translational rate of RPS6 mRNA, we processed polysomal fractions for qRT-PCR analysis. As expected, under unstimulated conditions, no significant differences were detectable in siSMN-transfected fibroblasts compared to siControl cells (Fig. 5b). Conversely, in stimulating conditions, RPS6 mRNA was significantly depleted from translating polysomes of SMN-deficient cells. As previously reported^14^, the ATP depletion/recovery assay recapitulates the actin-based membrane remodelling by coupling mTOR pathway activation with actin filament rearrangements. Thus, we checked mTOR signalling activation by western blot analysis. As shown in Fig. 5c, upon stimulation the p70S6 kinase, a downstream target of mTOR, shifted to an active status in both siControl- and siSMN-transfected fibroblasts, and this correlated with Ser235 and Ser236 phosphorylation in the RPS6 protein. This result demonstrates that SMN knockdown affects the translation rate of RPS6, despite the mTOR pathway switch on. In order to visualize newly synthesized RPS6 in single cells, we carried out a Puro-PLA assay, which couples puromycylation with a proximity ligation assay^49^. Subcellular distribution of PLA puncta, identifying newly synthesized RPS6, were then monitored by fluorescence microscopy (Fig. 5d). In unstimulated cells, PLA puncta were distributed throughout the cytoplasm, including sites close to the cell perimeter. A similar pattern has been found in both siControl- and siSMN-transfected fibroblasts. However, the frequency of PLA puncta in perinuclear regions was apparently higher in SMN-deficient cells. Interestingly, SMN deficiency perturbed RPS6 synthesis in stimulated cells. In stimulating conditions, SMN knockdown significantly decreased the number of PLA puncta (Fig. 5e), most of which appeared located in perinuclear regions. Together, these findings confirm that a subset of protein synthesis, occurring in an activity-dependent context, requires SMN function. Moreover, we propose that SMN could be implicated in ribosomal machinery assembly targeting ribosomal protein production in space and time.

### Caveolin-1 contacts RPS6 mRNA in an SMN-dependent manner

**Figure 6:**
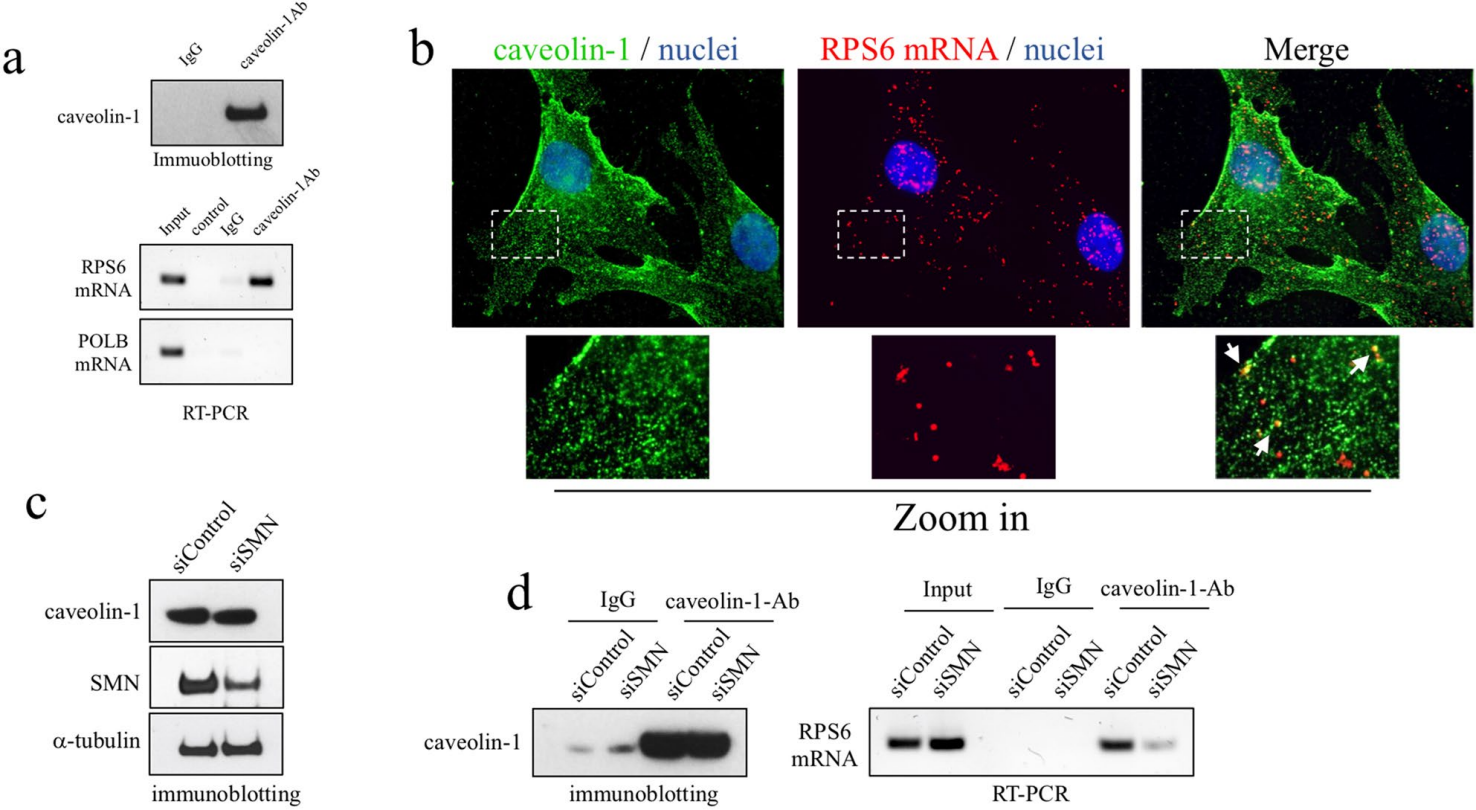
SMN mediates the association between caveolin-1 and RPS6 mRNA. (**a**) RIP assay in cellular extracts by using caveolin-1 antibody-conjugated beads or IgG- conjugated beads, as control. (bottom panel) RPS6 mRNA and DNA Polymerase Beta (POLB) mRNA in RIP samples was checked by semiquantitative RT-PCR analysed by agarose gel electrophoresis. (upper panel) Immunoblotting validating the efficiency of caveolin-1 immunoprecipitation. Panels are representative of three independent experiments. (**b**) Representative epifluorescence microscopy showing fibroblasts subjected to a combination of immunostaining for caveolin-1 protein (green) and a padlock assay targeting RPS6 mRNA (red). Nuclei were labelled with DAPI (blue). Scale bar, 10 µm. Overlapped fluorescent signals appear in yellow. Arrows in higher magnification of the boxed area indicate some overlapped dots. (**c**) Representative western blot analysis of the sample processed for RIP assay, validating the SMN depletion in siSMN-transfected fibroblasts, as well as the amount of caveolin-1. Alpha-tubulin was monitored as control of protein loading. (**d**) siControl- and siSMN-transfected fibroblasts were processed for RIP assay by using caveolin-1 antibody-conjugated beads or IgG- conjugated beads, as control. In the left panel is shown a representative western blot analysis validating the efficiency of caveolin-1 immunoprecipitation. In the right panel, the presence of RPS6 mRNA in immunoprecipitated material was checked by a semiquantitative RT-PCR analysed by agarose gel electrophoresis. RIP panels are representative of three independent experiments. All the uncropped gels/blots of figure are displayed in supplementary information.

We previously reported that SMN binds to and cooperates with caveolin-1 in promoting membrane-bound ribosomal proteins^14^. Based on this notion, we wanted to explore a potential link between caveolin-1 and transcripts encoding ribosomal proteins. To this end, we subjected fibroblasts to RIP experiments by using caveolin-1 antibody (Fig. 6a). Indeed, we showed that caveolin-1 complexes contain RPS6 transcript. To further support this result, we combined a padlock assay targeting RPS6 mRNA with an indirect immunofluorescence for caveolin-1 protein. Then, we imaged cells by fluorescence microscopy (Fig. 6b). Subcellular localization of caveolin-1 was coherent with its role as an integral protein of cellular membranes, included the plasma membrane. Consistent with the RIP results, caveolin-1 antibody partially overlapped with RCA dots generated by RPS6 padlock probes. To note, some of the overlapped dots appeared closely associated to plasma membrane domains. Next, we tested this novel interaction in a SMN deficiency context. We transfected fibroblasts with SMN1-selective siRNAs (siSMN) or scrambled siRNAs and (siControl) and processed cellular extracts for RIP experiments (Fig. 6c,d). Interestingly, SMN depletion impaired the association between RPS6 mRNA and caveolin-1. Through these findings we suggest that SMN could mediate the linkage between a subset of RP-coding mRNAs to caveolin-rich membrane compartments.

## Discussion

In eukaryotes, spatially restricted gene expression needs proper coordination between membrane dynamics, cytoskeleton tracks, and localized translation^5^. This widely shared mechanism implies that cell morphology can exacerbate unbalanced networks within the membrane dynamics-RNA metabolism axis. It is not surprising that defects in RNA-binding proteins, which act at the cross talk of the above cell processes, can become deleterious mainly in large and polarized cells^50^. Here, we provide further evidence that SMN protein, a key regulator of RNA metabolism, could compartmentalize protein synthesis within cells coupling the translation pathway and plasma membrane dynamics. Our findings point to a model by which SMN keeps localized distinct transcripts within cells by promoting anchoring of mRNPs. In particular, our study suggests that SMN could impact on ribosome biogenesis by spatial regulation of a subset of RP-coding transcripts.

It has been demonstrated that membrane trafficking orchestrates organelle-coupled protein synthesis, by supplying on-site translation machinery components^51^. In axons, late endosomes, which transport mRNPs, frequently pause on mitochondria where they serve as translation platforms^17^. Since endosomes move their content to and from the plasma membrane, we speculated that the plasma membrane could be a docking site for mRNA-containing complexes. Indeed, distinct regions of the plasma membrane sequester ribosomal proteins, which are released and recruited in translation hotspots upon specific stimuli. This event enables cells, such as neurons and fibroblasts, to rearrange the actin filament underlying cell surface plasticity^13,14^. To note, SMN deficiency depletes the plasma membrane of ribosomal proteins, and this correlates with the failure of fibroblasts to establish membrane polarity.

First, we confirmed the presence of ribosomal subunits and/or fully assembled ribosomes in membrane protrusions of fibroblasts, which actively rearrange the actin cytoskeleton. Next, to verify a coexistence of ribosomes and potential transcripts in membrane compartments, we carried out biochemical and imaging methodologies. We preliminarily focused on mRNAs encoding ribosomal proteins, due to their physical/functional link with SMN as well as their propensity to traffic toward the cell periphery^34,35,42–44^. We subjected fixed fibroblasts to a padlock assay, an alternative to single-molecule FISH methodology^45^. Fluorescence microscopy images revealed the presence of RPS6 mRNA diffusely throughout the cytoplasm and nuclear/perinuclear regions. Several RPS6 mRNA molecules were positioned in close proximity to the plasma membrane suggesting the existence of membrane-bound RPS6 transcripts. We validated this result also by biochemical analysis of plasma membrane-enriched fractions (PMEFs) isolated from fibroblasts. In our system, we found that SMN deficiency perturbs subcellular compartmentalization of RPS6 mRNA. A reduced level of SMN protein induces a nuclear/perinuclear accumulation of RPS6 mRNA, and, most importantly, reduces membrane compartmentalization of RPS6 transcripts.

These findings highlight the possibility that subcellular sequestering of RPS6 transcript could regulate its expression levels both in time and space. Our hypothesis is that membrane-bound RPS6 transcripts might undergo two different fates. Newly synthesized RPS6 could supply translation machinery components implicated in peripheral protein synthesis. In alternative, RPS6 mRNA might undergo endocytosis and endosomal trafficking. To note, late endosomes can act as hotspots for local translation onto the mitochondrial membrane^17^. In each case, membrane networks seem to work in intimate connection with the translation machinery control. Nuclear accumulation of RPS6 mRNA in SMN-deficient fibroblasts remains instead enigmatic. A widely accepted notion is that in mammalian cells, mature transcripts are mostly localized in the cytoplasm. However, the existence of mRNA nuclear pools has been reported in previous studies. Gondran et al., identified several genes whose mature mRNA may be detected in the nucleus independently of their expression level^52^. They found that the ratio of cytoplasmic to nuclear mRNA accumulation varies between genes and cell types. Didiot et al. explored the subcellular localization of Huntingtin (Htt) mRNA in non-neuronal and neuronal cells^53^. Htt transcript is detected more often in the nuclear compartment than in the cytoplasm of neuronal cells, suggesting that a nuclear retention of mRNAs could become exacerbated in cell with a large and polarized surface. It is plausible that nuclear compartmentalization of mRNAs impacts RNA metabolism as well as gene expression control. Our hypothesis is that newly transcribed RPS6 mRNA accumulate in nuclei to prevent deleterious translation events.

Notably, SMN deficiency affects not only subcellular localization of RPS6 mRNA but also its translation rate under dynamic actin/membrane rearrangements. This observation fits in part with a recent study showing a perturbed translation efficiency of RP-coding transcripts in motor neurons from a mouse model of SMA^44^. Moreover, we found also the association of SMN protein with RP-coding transcripts, as previously indicated^34^.

Finally, we provide evidence that caveolin-1, an integral membrane protein, associates with RP-coding transcripts in a SMN-dependent manner. It is noteworthy that SMN deficiency imposes similar changes in both RPS6 transcript and its encoding protein^14^. Again, our studies highlight a critical interplay between membrane dynamics and SMN-mediated translational control.

Interestingly, our data converge on an intriguing issue concerning ribosome biogenesis. Ribosome biogenesis is a cellular event of extraordinary complexity^54,55^. An accepted notion is that assembly of ribosomal subunits occurs in the nucleus/nucleolus. Following their synthesis in the cytoplasm, RPs are imported into the nucleus, assembled with the rRNA in the nucleolus, and re-exported into the cytoplasm, where they form a mature and functional ribosome. So, it is unclear why many cell types need to allocate a subset of RP-coding mRNAs far from perinuclear subregions^35,42,43^. One hypothesis would be that structural components of translation machinery are themselves locally synthesized. Itzkovitz and co-workers, suggest a model by which transcripts encoding RPs are located in the less translationally active basal side of the intestinal epithelium in fasting mice. Upon refeeding, these transcripts move toward the more translationally active apical cell side. The polarized trafficking of RP-coding transcripts correlates with a rapid increase in their translational efficiency^35^. Therefore, a cellular strategy to perform specialized functions seems to be to invest resources in making first the “machine” and then using it to produce appropriate factors. Recently, Holt and co-workers showed that the ribosome structure may undergo to dynamic changes through a nucleolus-independent mechanism^56^. The authors suggest that extrinsic signals can trigger axonal production of RPs that dynamically modify the ribosome machinery for axon branching and synaptogenesis.

Local translation of ribosomal proteins could be indicative of extra-ribosomal functions^57^ or, most intriguingly, “specialized ribosomes”^58,59^. As strongly sustained by Barna’s laboratory, ribosome heterogeneity, resulting from rRNA diversity and differential expression/post-translational modifications of ribosomal proteins, may provide a crucial new layer for the spatiotemporal control of gene expression^58,59^. In addition, cells can adapt the stoichiometry among core ribosome components depending on physiological conditions^60^. Thus, a local specialized proteome may be achieved by altering ad hoc ribosomal subunit composition. Our opinion is that intracellular sites wherein distinct transcripts are recruited may dictate their differential expression and, thus, distinctive activities of their encoding proteins. In this study we highlighted for the first time the ability of the plasma membrane to retain mRNAs, in particular, RP-coding transcripts. As a consequence, the plasma membrane could keep and govern a specialized translation machinery in order to support its activities. Since SMN may be involved in membrane compartmentalization of RP-coding transcripts, we hypothesize that SMN could promote specialized translation underlying membrane plasticity. In this regard, it is important to mention that the dysregulation of neuromuscular junction (NMJ) may be an early event in SMA pathogenesis. Coherently, abnormalities of NMJ precede the motor neuron degeneration in SMA patients^61^. Notably, NMJ is a highly specialized plasma membrane domain by which motor neuron communicates with muscle. Furthermore, proper NMJ morphology/plasticity requires activity-dependent local translation^62^. Again, the molecular context in which SMN appears strongly implicated includes plasma membrane-related networks.

Our ongoing studies aim to (1) obtain a complete list of RP-coding transcripts associated to membrane compartments; (2) identify a potential transcriptional profile of plasma membrane- enriched fractions, and (3) reveal changes occurring in SMN deficiency. In addition, our major challenge is to understand how plasma membrane domains sequester mRNAs. Even if ribosomes and mRNAs coexist in membrane domains, at this stage we cannot explain whether membrane-bound mRNAs are engaged in monosomes and/or polysomal complexes. Further investigations are needed to bridge this critical gap.

## Methods

### Antibodies and reagents

The following antibodies were used: anti-ribosomal RNA monoclonal antibody, clone Y10B was a gift from Dr. Christian Barbato (CNR-IBBC, Italy), work dilution for immunofluorescence, 1:100; anti-SMN mouse monoclonal antibody (cat. no. 610647, BD Transduction Laboratories; work dilution for western blotting, 1:10,000; for immunofluorescence, 1:150); anti-S6 rabbit polyclonal antibody (cat. no. 2217, Cell Signaling Technology; work dilution for western blotting 1:1000; for immunofluorescence 1:200); anti-phospho-S6 (Ser235 or Ser236) rabbit monoclonal antibody (cat. no. 4858, Cell Signaling Technology; work dilution for western blotting 1:1000); anti-phospho-p70S6K (Thr389) rabbit polyclonal antibody (cat. no. 9234, Cell Signaling Technology; work dilution for western blotting 1:1000); anti-glyceraldehyde‐3‐phosphate dehydrogenase (GAPDH) mouse monoclonal antibody (cat. no. G8795; work dilution for western blotting 1:5000); anti-α-tubulin mouse monoclonal antibody (cat. no. T6074, Sigma-Aldrich; work dilution for western blotting, 1:2000); anti-Caveolin-1 rabbit polyclonal antibody (cat. no. sc-894, Santa Cruz Biotechnology; work dilution for western blotting, 1:5000); anti-Caveolin-1 mouse monoclonal antibody (cat. no. MAB5736, R&D Systems; work dilution for immunofluorescence, 1:200); anti-puromycin mouse monoclonal antibody (cat. no. MABE343, Millipore; work dilution for western blotting, 1:25,000; for immunofluorescence, 1:10,000). The secondary antibodies conjugated to horseradish peroxidase were purchased from Jackson ImmunoResearch Laboratories and used at a dilution of 1:10,000. The Alexa Fluor488-conjugated secondary antibodies were purchased from Thermo Fisher and were used at a dilution of 1:250. The Alexa Fluor594-conjugated phalloidin were from Thermo Fisher Scientific, Inc., Waltham, MA, USA.

### Cell cultures and transfection

hTert-immortalized human fibroblasts (hTert-Fibroblasts) were obtained from Silvia Soddu (Regina Elena Cancer Institute, Italy)^63^. As described previously^14^, hTert-Fibroblasts were cultured in Dulbecco’s modified Eagle’s medium (DMEM, Gibco), supplemented with heat inactivated 10% FBS (Australian, Gibco), penicillin-streptomycin (Gibco) and GlutaMAX (Gibco), in a 5% CO2 humidified atmosphere, at 37 °C. Human fibroblasts from SMA type I patient (GM00232) and healthy control (GM08333) were obtained from Coriell Institute for Medical Research (Camden, NJ, USA), and cultured in DMEM medium supplemented with 10% FBS, penicillin/streptomycin, and GlutaMAX, in 5% CO2 humidified atmosphere, at 37 °C. For knockdown experiments, cells were transfected with Lipofectamine 2000 (Thermo Fisher Scientific) and a combination of three siRNA-27 duplexes targeting the human SMN1 gene (OriGene), following manufacturer’s instructions. Universal scrambled siRNA duplex was used as negative control. Cells were harvested after 48 h or 72 h post transfection.

### ATP depletion and recovery assay

The ATP depletion/recovery assay was performed as described previously^14,47^. Briefly, fibroblasts were incubated in PBS supplemented with 1 mM CaCl_2_, 1 mM MgCl_2_ and 20 mM NaN_3_, for 1 h. This treatment produces a rapid depletion of the cellular ATP, triggering a reversible disassembly of actin filaments. NaN_3_-containing buffer was then replaced with fresh medium supplemented with heat inactivated 10% FBS (Australian, Gibco), for 30 min, allowing ATP recovery. Rapid restoration of actin cytoskeleton occurred as a synchronous burst of membrane protrusions.

### Preparation of plasma membrane-enriched fractions

Plasma-membrane-enriched fractions (PMEFs) was isolated as previously described^14^. Briefly, cells were lysed in buffer A (5 mM Tris–HCl pH 7.4, 1 mM EGTA, 1 mM DTT and 320 mM sucrose). Extracts were passed through a 26G needle five times and centrifuged at 1000 g for 10 min at 4 °C. The supernatant was kept, and the pellet was quickly vortexed in the presence of original volume of lysis buffer and centrifuged at 1000 g for 10 min at 4 °C. The two supernatants were pooled and centrifuged at 24,000 g for 20 min at 4 °C in a Beckman SW41 rotor. The supernatant was discarded, and the pellet was resuspended in 12 ml of buffer B (5 mM Tris–HCl pH 7.4, 1 mM EGTA and 1 mM DTT), and centrifuged at 24,000 g for 30 min at 4 °C in a Beckman SW41 rotor. The supernatant was discarded. The pellet was aliquoted and processed for both RNA and protein extractions.

### Immunofluorescence analysis

Immunofluorescence analysis was performed as described previously^14^. Briefly, cells were fixed with 4% formaldehyde in PBS, permeabilized in 0.2% Nonidet P40 (Boehringer Mannheim) for 20 min and blocked with 1% BSA in PBS at room temperature. Samples were incubated with the primary antibodies, washed three times in PBS and then incubated with the appropriate secondary antibodies. Slides were mounted with ProLong with Dapi (Thermo Fisher Scientific) and examined by a conventional epifluorescence microscope (Olympus BX53; Milano, Italy). Images were captured by a SPOT RT3 camera and elaborated by IAS software.

### RNA Immunoprecipitation (RIP) assay

Cells were resuspended in IP Buffer (50 mM Tris–HCl pH 7.5, 250 mM NaCl, 5 mM EDTA, 50 mM NaF, 0.1 mM NaVO_4_, 0.1% Triton X-100, 5% glycerol and complete protease inhibitor cocktail (Roche)), in the presence of RNase inhibitors (Thermo Fisher Scientific). Extracts were vortexed 3 times for 10 s, incubated in ice for 20 min and centrifuged at 10,000 g for 7 min at 4 °C. For the immunoprecipitation assay, the protein lysate was pre-cleared with Protein A/G-Agarose beads (Roche, Indianapolis, IN, USA), pre-saturated in 2% BSA-PBS, by replacing beads 3 time within 90 min, at 4 °C. Then 1200 µg of extract was immunoprecipitated in IP buffer overnight with the anti-SMN monoclonal antibody or with anti-caveolin-1 polyclonal antibody. As negative control, the immunoprecipitation was carried out with mouse IgG-beads. The beads were washed five times for 5 min at 4 °C in IP buffer and once in PBS buffer. The immunoprecipitated samples were resuspended in IP buffer. A portion of immunoprecipitation was analysed by western blot analysis. RNA was extracted using Qiazol reagent (Thermo Fisher Scientific), according to the manufacturer’s instructions. RNAs were converted to cDNAs using a High Capacity cDNA Reverse Transcription kit (Thermo Fisher Scientific).

### RNA extraction, retrotranscription and quantitative real-time PCR (qRT-PCR)

RNA from whole cell extract and PMEF fraction of fibroblasts was extracted using Qiazol reagent according to the manufacturer’s instructions and was then reverse transcribed using a High Capacity cDNA Reverse Transcription kit (Thermo Fisher Scientific). Quantitative real-time PCR (qRT-PCR) assay was performed in triplicate in a 96-well format in an ABI Prism 7000 Sequence Detection System (Applied Biosystems, Foster City, CA, USA) using the SYBR Green PCR Master mix. Relative transcript expression was calculated using the comparative Ct method (2^-ΔΔCt^). ΔCt is calculated as the difference between the Ct for the target transcript and the Ct for the geometric mean of GAPDH and beta-Actin. ΔΔCt is the result of the difference between the ΔCt for the siSMN sample and the ΔCt for the siControl sample or between the ΔCt for the SMA type I sample and the ΔCt for the unaffected sample.

Primer sequences used in this study are shown in additional file: Table S1.

### Phosphorylation of the padlock probe

The phosphorylation of padlock probe was carried out in a volume of 20 µl by adding: 2 µl of the linear padlock probe (100 µM), 2 µl of 10X T4 polynucleotide kinase reaction buffer, 0.5 µl of T4 polynucleotide kinase (10 U µl^−1^) and 15.5 µl of DEPC-treated H_2_O. Reaction was incubated for 30 min at 37 °C, 10 min at 42 °C and finally 10 min at 65 °C.

### Padlock assay

Cells were fixed with 4% formaldehyde in PBS, permeabilized in 0.2% Nonidet P40 (Boehringer Mannheim) for 20 min and then incubated overnight at 37 °C with the specific padlock probe to the target mRNA. The reaction was conducted in 20 µl mixture containing 2 µl of phosphorylated padlock probe (10 µM), 1 µl of DTT (100 mM), 0.5 ul of RiboLock RNase Inhibitor (40 U µl^−1^) and 16.5 µl of DEPC-treated H_2_O. The sample was washed twice for 5 min at room temperature in PBS/0.01% Tween-20. Then cells were incubated for 2 h at 37 °C with the circularization reaction mixture (1X SplintR ligase buffer, 2.5 U µl^−1^ Splint R ligase and 1 U µl^−1^ RiboLock RNase Inhibitor). After a 5 min wash in PBS/0.01% Tween-20, the RCA primer mixture (0.2 µM RCA primer, 1X SSC, 10% formamide, 5 mM DTT and 0.5 U µl^−1^ RiboLock RNase Inhibitor) was added to the sample and incubated for 1 h at 37 °C. RCA reaction was then carried out for 2 h at 37 °C, the sample was incubated with 10 µl of a mixture containing 1 µl of 10X phi29 DNA polymerase reaction Buffer, 3 µl of dNTPs(10 mM of each dATP, dCTP, dGTP and dTTP), 0.5 µl di phi29 DNA polymerase (10 U µl^−1^), 0.25 µl of RiboLock RNase Inhibitor (40 U µl^−1^) and 5.25 µl of DEPC-treated H2O. The incubation was followed by two washes of 5 min each in PBS/0.01% Tween-20. Finally, RCA amplicons were detected by 100 nM fluorophore-labelled detection probe in 2X SSC and 15% formamide for 30 min at 37 °C. After three washes in PBS/0.01% Tween-20 for 5 min each, slides were mounted or further processed for immunofluorescence analysis, as described above^14^. High-resolution (100 × objective) images were analysed by ImageJ (National Institutes of Health) to calculate the density of amplicon dots.

### Puro-PLA assay

Cells were incubated with 10 µM puromycin in the presence of 355 µM Cycloheximide (CHX, Sigma-Aldrich) for 10 min. After a briefly wash in PBS supplemented with 355 µM CHX, cells were incubated in the fixative/extraction buffer (4% formaldehyde, 0.015% digitonin, 355 µM CHX, 1X PBS) for 20 min at room temperature and then permeabilized in 0.2% Nonidet P40 for 20 min. Therefore, samples were subjected to in situ PLA using Duolink In Situ Detection Reagent Green Kit (DUO92008 Sigma-Aldrich), according to the manufacturer’s instructions. A combination of primary antibodies to puromycin (mouse monoclonal antibody) and ribosomal protein S6 (rabbit polyclonal antibody) was used. PLA signal was detected by epifluorescence microscope (Olympus BX53; Milano, Italy). High-resolution (100 × objective) images were analysed by ImageJ (NIH) to calculate the density of PLA puncta.

### Polyribosome profiling

Cells were homogenized in lysis buffer (10 mM Tris–HCl pH 7.5, 100 mM NaCl, 10 mM MgCl_2_, 1% Triton X-100, 30 U/ml RNasin). Lysates were incubated on ice for 5 min and then centrifuged at 12,000 rpm for 5 min at 4 °C. Supernatants were immediately loaded onto a 10 ml 15–50% (w/v) sucrose gradient and centrifuged at 37,000 rpm for 180 min at 4 °C in a Beckman SW41 rotor. Polysomes, 80S monosome, the two free ribosomal subunits 60S and 40S as well as the light mRNPs were detected by UV absorbance at 254 nm using a BioLogic LP system (BioRad Inc., Hercules, CA, USA). Each gradient was collected in 9 fractions, and the proteins were precipitated with a mix containing 50% ethanol, 25% methanol and 25% acetone and were then processed for western blot analysis. For RPS6 mRNA analysis, fractions 6–9 (polysomal fraction) were pooled and quantitative real-time PCR (qRT-PCR) was performed. GAPDH was used for the normalization of mRNA, and the relative expression was calculated using the comparative Ct method (2^-ΔΔCt^).

### Quantification and statistical analysis

All experiments were performed on at least three independent biological replicates. Data are presented as mean ± s.d. Statistical analysis was performed using the GraphPad Prism software. Data were analysed using unpaired t-test or one-way ANOVA test with Bonferroni test for multiple comparison as specified in the figure legends; *P* < 0.01 was considered statistically significant.

Additional analysed data files are available in the Supplementary Information of this article.

## Supplementary information

Supplementary Information.
