# Supplementary material for: SMN protein promotes membrane compartmentalization of ribosomal protein S6 transcript in human fibroblasts
Source: Sci Rep. 2020 Nov 4;10:19000. doi: 10.1038/s41598-020-76174-3 (PMC7643083; doi:10.1038/s41598-020-76174-3)
Supplement: Supplementary file 1 — Supplementary Information. [file 41598_2020_76174_MOESM1_ESM.pdf]

SMN protein promotes membrane compartmentalization of ribosomal protein S6 transcript in human fibroblasts.

Francesca Gabanella[1,2*], Annalisa Onori[2], Massimo Ralli[3], Antonio Greco[3], Claudio Passananti[2], Maria Grazia Di Certo[1*].

1 CNR-Institute of Biochemistry and Cell Biology, Department of Sense Organs, Sapienza University of Rome, Viale del Policlinico, 155-00161 Rome, Italy.

2 CNR-Institute of Molecular Biology and Pathology, Department of Molecular Medicine, Sapienza University of Rome, Viale Regina Elena, 291-00161 Rome, Italy.

3 Department of Sense Organs, Sapienza University of Rome, Viale del Policlinico, 155-00161 Rome, Italy.

*To whom correspondence should be addressed

e-mails: mariagrazia.dicerto@cnr.it; francesca.gabanella@gmail.com

Table S1

Table S1

Oligos used in this study

Primer name	Primer sequence (5'-3')
RT-PCR GAPDH F	CATGAGAAGTATGACAACAGCCT
RT-PCR GAPDH R	AGTCCTTCCACGATACCAAAGT
RT-PCR BETA-ACTIN F	CATGTACGTTGCTATCCAGGC
RT-PCR BETA-ACTIN R	CTCCTTAATGTCACGCACGAT
RT-PCR POLB F	GTGAGACAAAGTTCATGGGTGT
RT-PCR POLB R	GTGAAACCCTTTTCTAGGGCAT
RT-PCR RPS6 F	AAGAGCTAGCAGAATCCGCA
RT-PCR RPS6 R	GCAGGACACGTGGAGTAACA
Padlock Probe RPS6	CACCACTGATTCGGATTTTTTTCTCAATTCTGCTACTTTACTACC TCAATTCTGCTACTGTACTACTTTTTTCCTTGTTTGTCGTTCC
RCA Primer	AGTACAGTAGCAGAATTGAG
AlexaFluor 595-labelled probe	CTCAATTCTGCTACTTTACTAC

Supp. Figure S1. SMN knockdown increases the RPS6 transcript amplicons. (a) Representative images of siControl- and siSMN-transfected fibroblasts. Cells were immunostained for SMN (green) and subjected to a padlock assay targeting RPS6 mRNA (red dots) Nuclei were labelled with DAPI (blue). Scale bar, 10 µm. (a) Padlock imaging for RPS6 mRNA amplicons (white dots) in siControl and siSMN-transfected fibroblasts. Scale bar 10 µm. (b) Graph illustrates the number of RPS6 mRNA amplicons in siControl and siSMN-transfected fibroblasts (n=50 cells were analysed for each condition). Asterisks indicate significative differences using unpaired t-test (**** P < 0.01; *P value* < 0.0001). Data are the means of three independent experiments. Error bars indicate s.d..

Supp. Figure S2. Validation of plasma membrane-enriched fractions. Western blot analysis of whole cell extracts (WCE) and plasma membrane-enriched fractions (PMEFs) from siControl and siSMN-transfected fibroblasts. Equal amounts of the protein extracts were immunoblotted for SMN and caveolin-1. Uncropped blots are displayed in supplementary information.

Supp. Figure S3. Reduced membrane compartmentalization of RPS6 transcripts in SMA type I fibroblasts. (a) Validation of plasma membrane-enriched fractions in unaffected and SMA type 1 (SMA I) human primary fibroblasts by western blot analysis. Equal amount of protein extracts was immunoblotted for SMN and caveolin-1. Uncropped blots are displayed in supplementary information. (b) Quantification of RPS6 transcript by RT-PCR. For each experimental condition (unaffected and SMA type 1 human primary fibroblasts) both the whole cell extract (WCE) and plasma membrane fraction (PMEF) were assessed. Results are shown as fold change comparing SMA type 1 fibroblasts (SMA I) with unaffected fibroblasts. Asterisks indicate significative differences using unpaired t-test (*** P < 0.01; WCE *P value* < 0.0001; PMEF *P value* = 0.0002) Data are the mean from three independent experiments. Error bars indicate s.d..

Supp. Figure S4. SMN affects translation efficiency of RPS6 mRNA. Polysome profile of the siControl and siSMN-transfected fibroblast, unstimulated or stimulated by 1hour ATP-recovery. In the graph are compared the UV absorbance profile at 254 nm of the cytosolic extracts separated through sucrose density gradient. Nine fractions from the top to the bottom of the gradient were collected and subjected to western blot analysis. The material corresponding to mRNPs, 40S/60S subunits, 80S monosomes and polysomes are indicated. Each fraction was tested for distribution of SMN protein. Ribosomes was checked by RPS6. Uncropped blots are displayed in supplementary information.

Supplementary figure S1

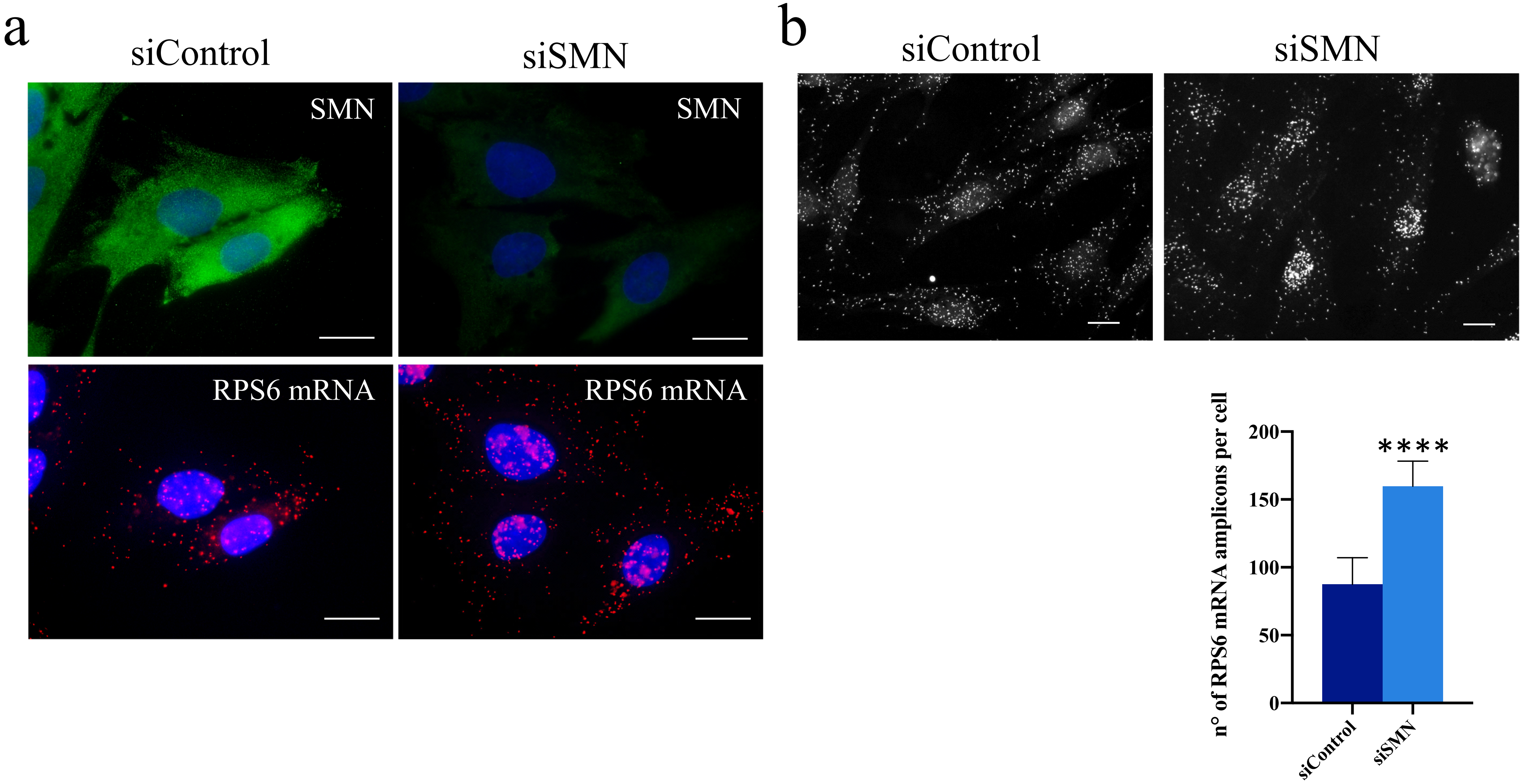

Supplementary figure S2

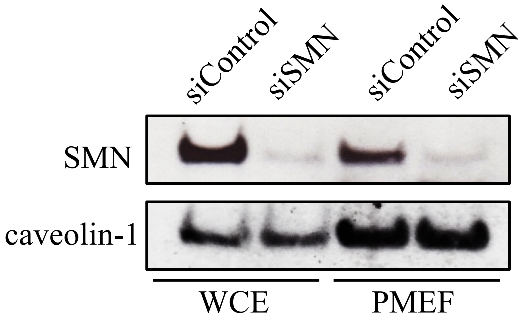

Supplementary figure S3

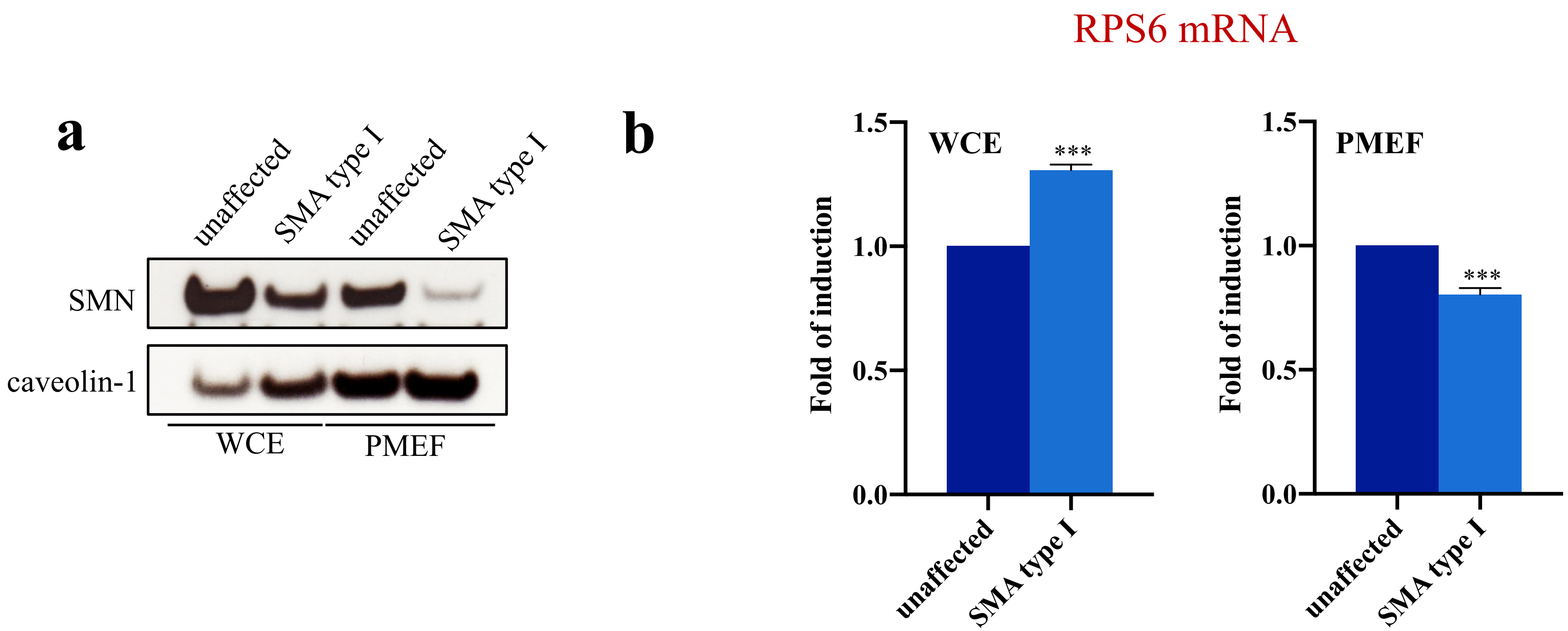

Supplementary figure S4

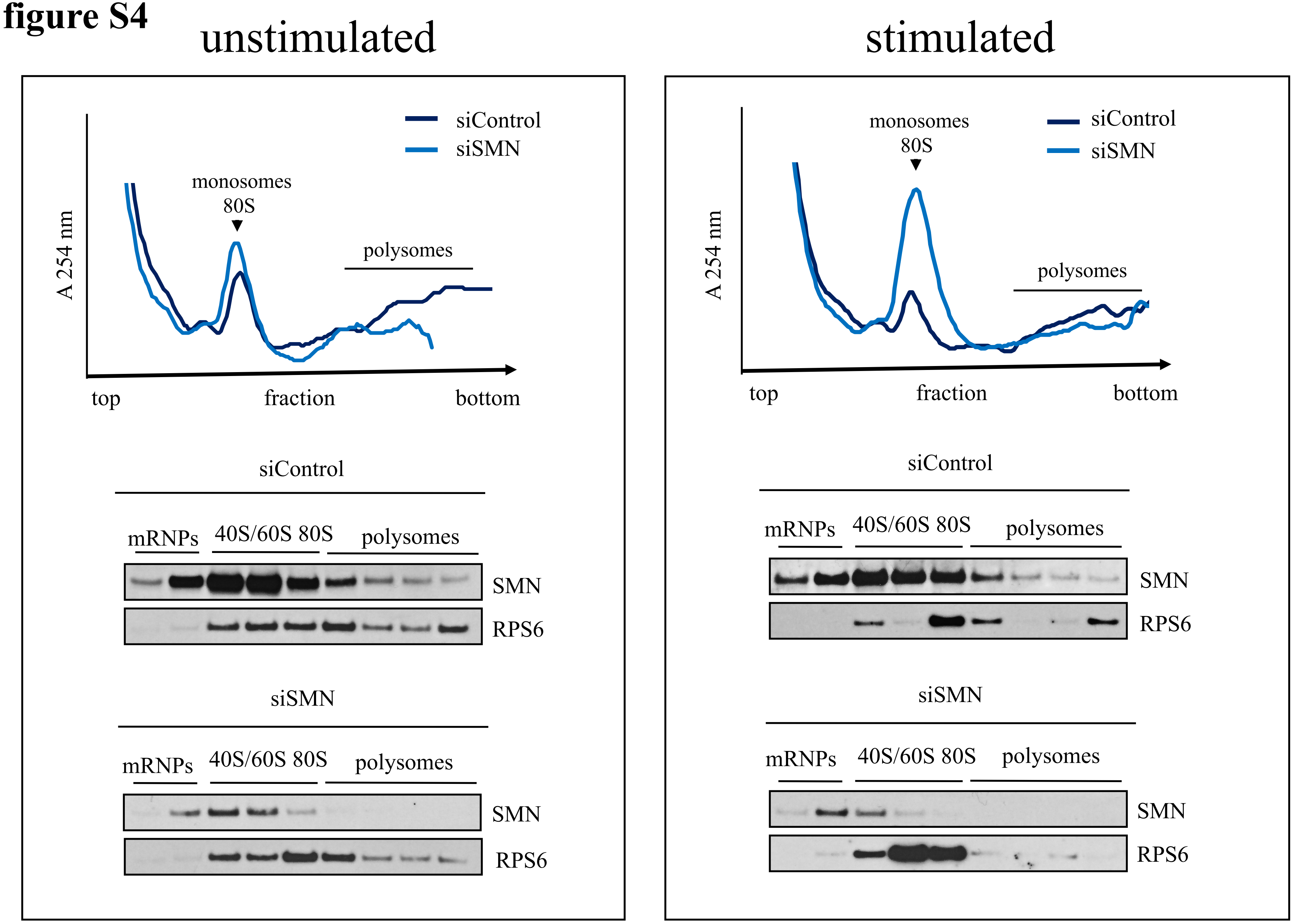

Supplementary figure 5

Original uncropped immunoblotting from figure 4a.

SMN

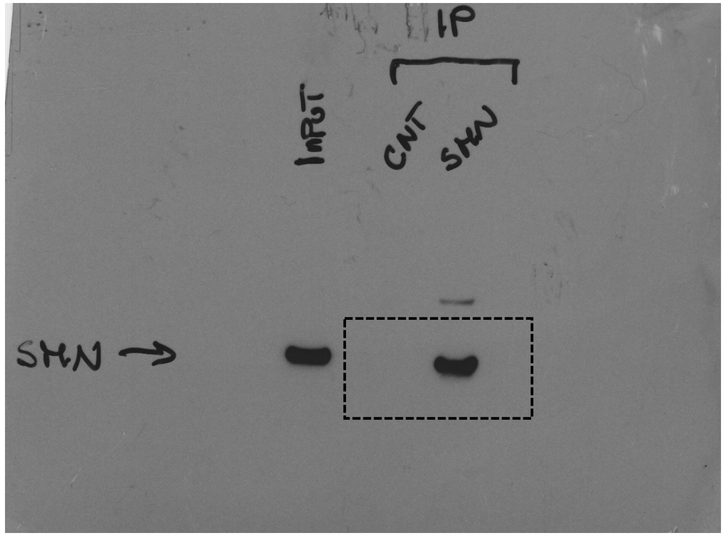

Original uncropped gel from figure 4a

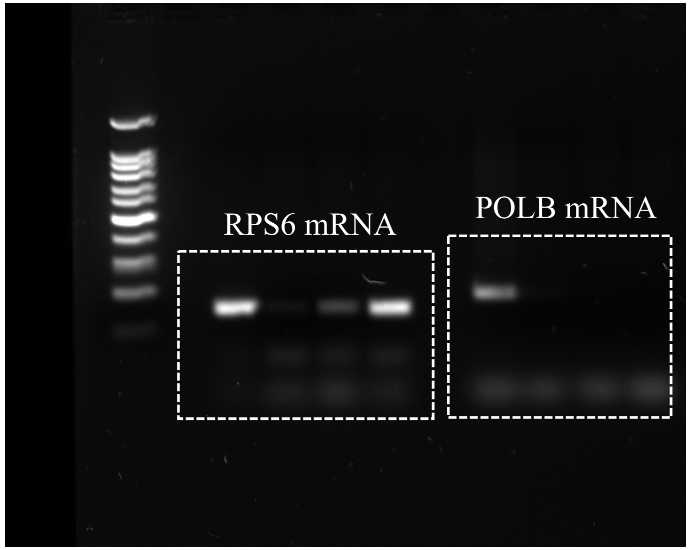

Inverted uncropped gel from figure 4a

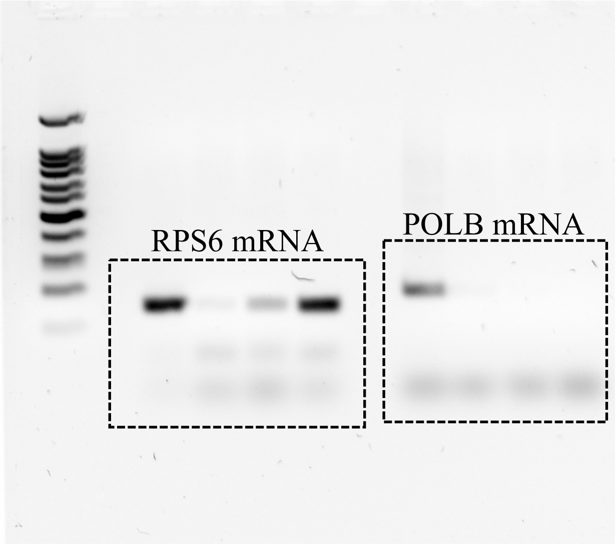

Supplementary figure 6

Original uncropped immunoblotting from figure 5c.

ph-p70S6K

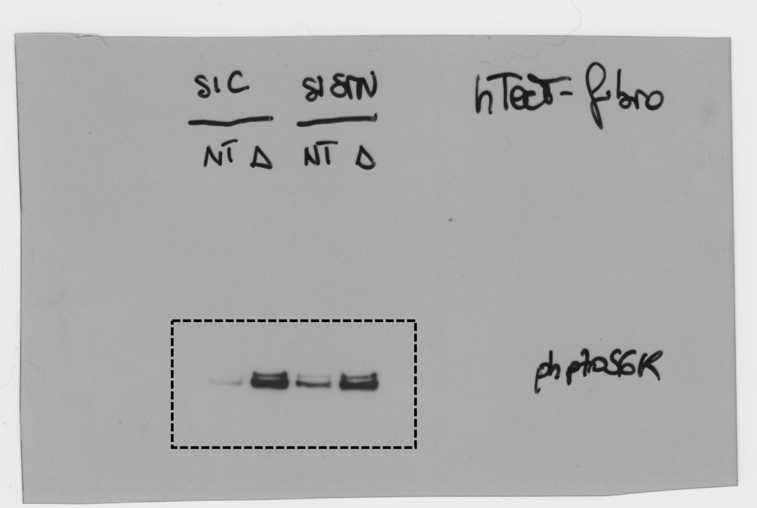

ph-RPS6

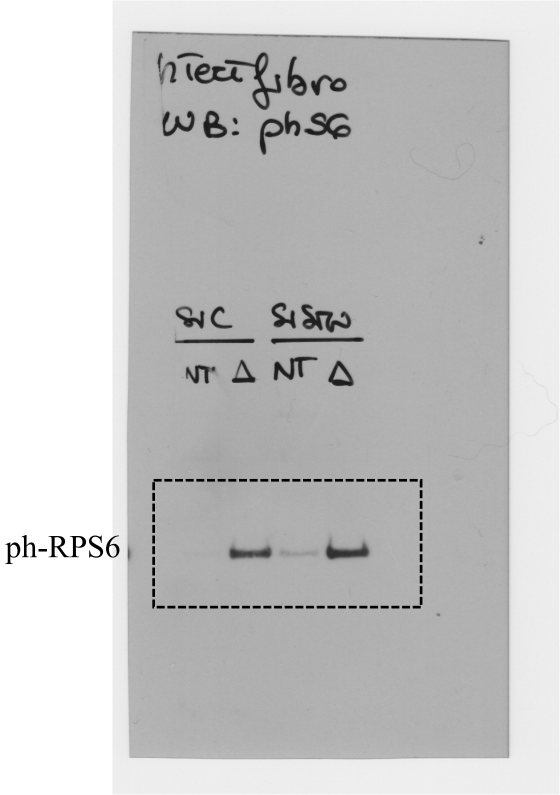

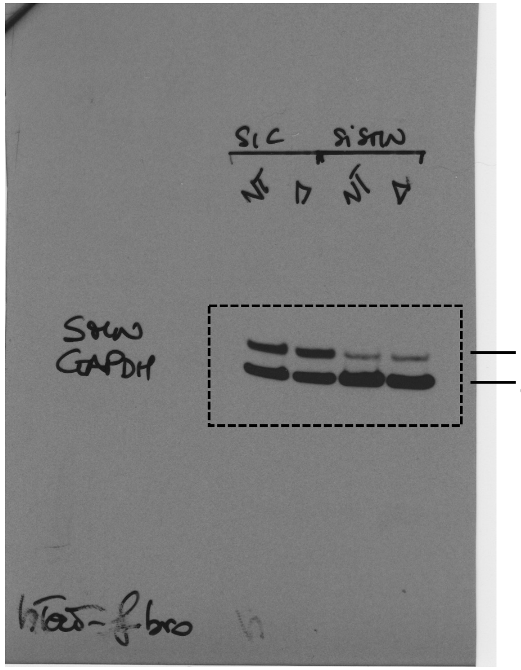

SMN
GAPDH

Supplementary figure 7

Original uncropped immunoblotting from figure 6a.

caveolin-1

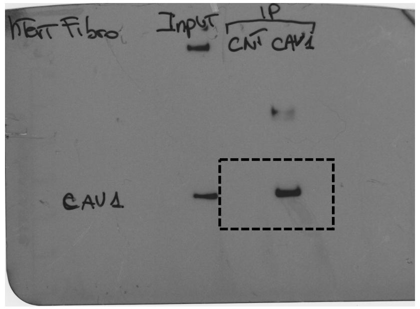

Original uncropped gel from figure 6a

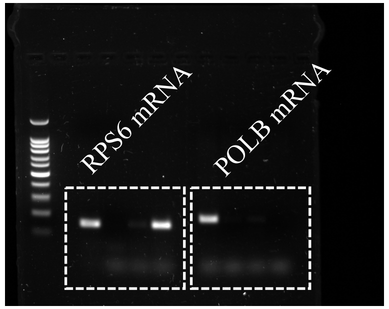

Inverted uncropped gel from figure 6a

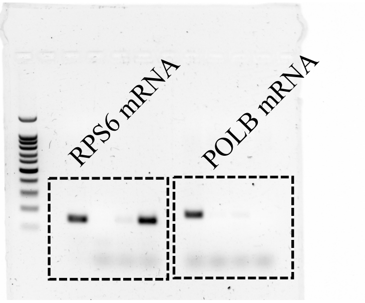

Original uncropped immunoblotting from figure 6c.

caveolin-1

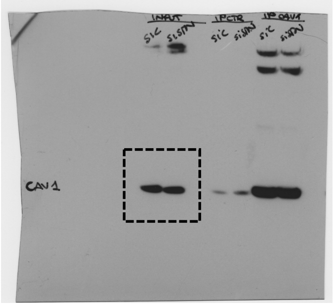

SMN

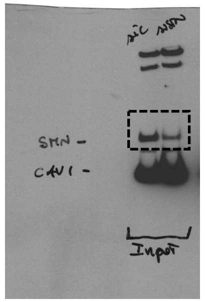

α-tubulin

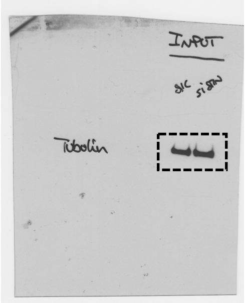

Original uncropped immunoblotting from figure 6d.

caveolin-1

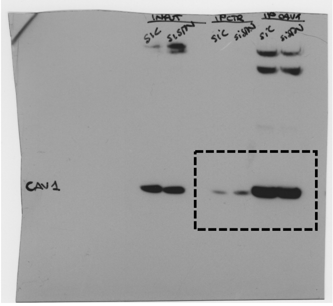

Original uncropped gel from figure 6d

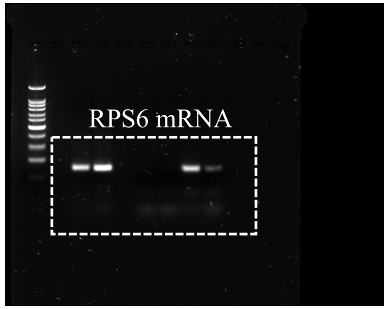

Inverted uncropped gel from figure 6d

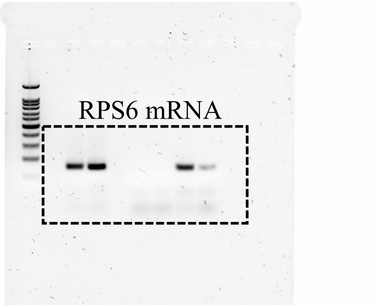

Supplementary figure 8

Original uncropped immunoblotting from figure S2.

SMN

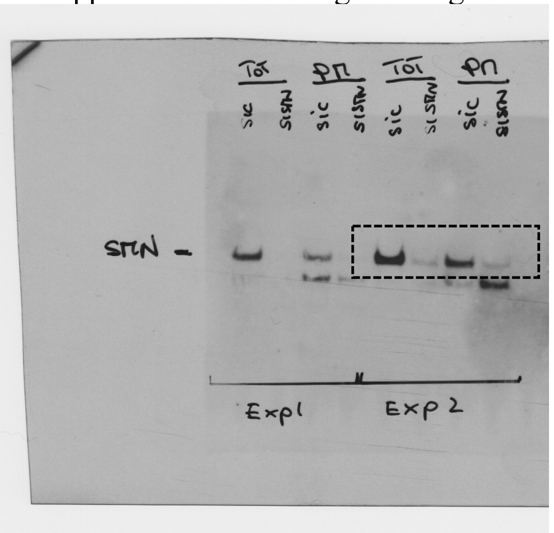

caveolin-1

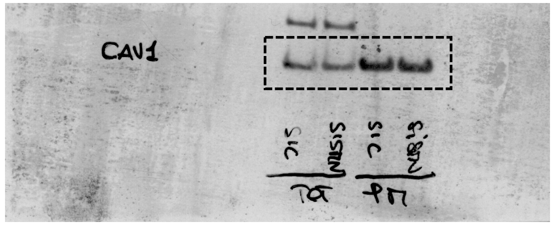

Supplementary figure 9

Original uncropped immunoblotting from figure S3.

SMN

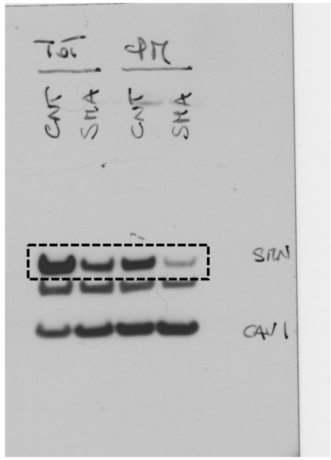

caveolin-1

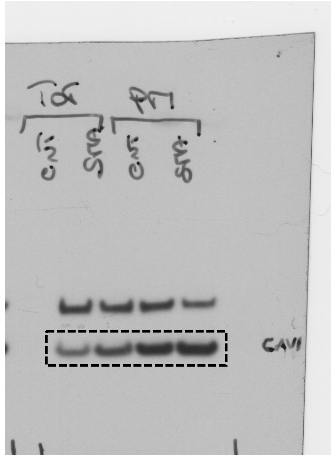

Supplementary figure 10

Original uncropped immunoblotting from figure S4.

SMN

RPS6

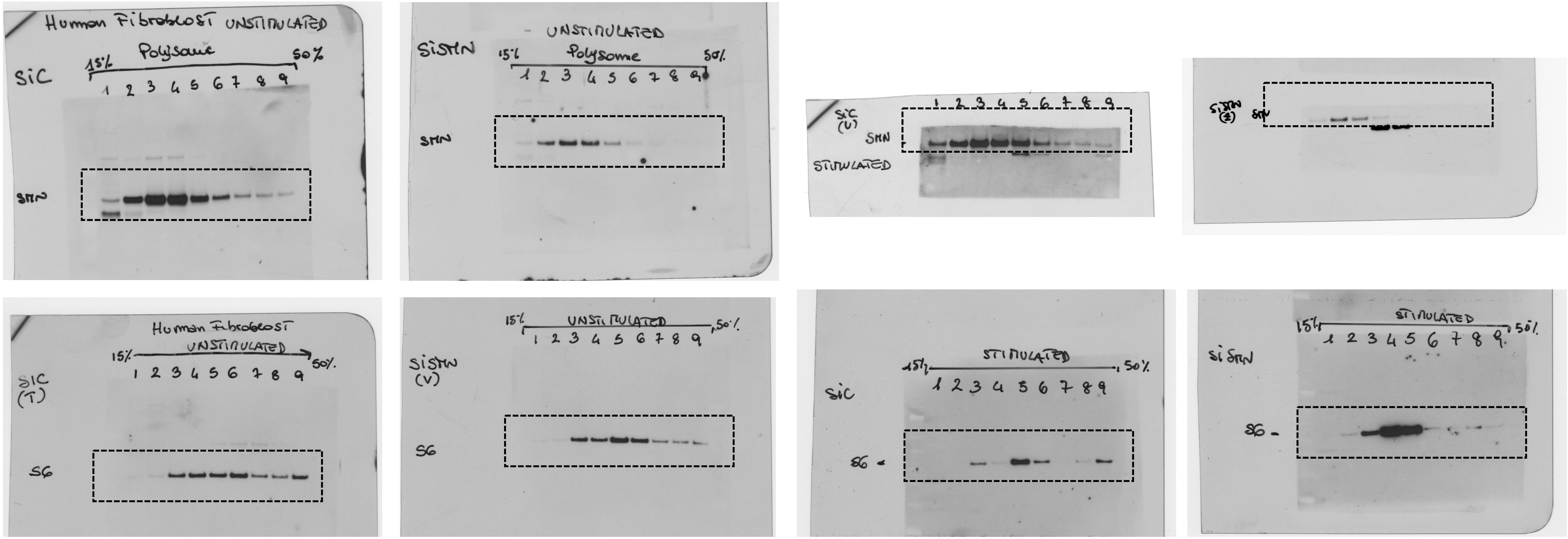

siControl unstimulated

siSMN unstimulated

siControl stimulated

siSMN stimulated
